# “It means so much for me to have a choice”: a qualitative study providing first-person perspectives on medication-free treatment in mental health care

**DOI:** 10.1186/s12888-020-02770-2

**Published:** 2020-08-08

**Authors:** Christine H. Oedegaard, Larry Davidson, Brynjulf Stige, Marius Veseth, Anne Blindheim, Linda Garvik, Jan-Magne Sørensen, Øystein Søraa, Ingunn Marie Stadskleiv Engebretsen

**Affiliations:** 1 Haukeland University Hospital, Kronstad DPS, Pb 1400, 5021 Bergen, Norway; 2 Centre for International Health, Department of Global Public Health and Primary Care, University of Bergen, Pb. 7804, 5020 Bergen, Norway; 3 Department of Psychiatry, Yale Medical School, New Haven, Connecticut USA; 4 The Grieg Academy, University of Bergen, Pb. 7800, 5020 Bergen, Norway; 5 Department of Clinical Psychology, University of Bergen, Pb. 7807, 5020 Bergen, Norway; 6 Hvite Ørn User Organisation, Thomles gt. 4, 0270 Oslo, Norway

**Keywords:** Recovery, Communication, Decision making, Lived experience, Psychosis, Medication, Quality of care

## Abstract

**Background:**

In 2016, the Western Norway Regional Health Authority started to integrate more evidence-based psychosocial interventions into the existing mental health care, emphasizing the right for persons with psychosis to choose medication-free treatment. This change emerged from the debate on the effectiveness and adverse effects of the use of antipsychotic medication. Aspects beyond symptom reduction, such as interpersonal relationships, increased understanding of one’s own pattern of suffering, hope and motivation, are all considered important for the personal recovery process.

**Methods:**

This study explores whether these aspects were present in users’ descriptions of their recovery processes within the medication-free treatment programme in Bergen, Western Norway. We interviewed ten patients diagnosed with psychosis who were eligible for medication-free services about their treatment experiences. Data were analysed using Attride-Stirling’s thematic network approach.

**Results:**

The findings show a global theme relating to personal recovery processes facilitated by the provision of more psychosocial treatment options, with three organizing subthemes: interpersonal relationships between patients and therapists, the patient’s understanding of personal patterns of suffering, and personal motivation for self-agency in the recovery process. Participants described an improved relationship with therapists compared to previous experiences. Integrating more evidence-based psychosocial interventions into existing mental health services facilitated learning experiences regarding the choice of treatment, particularly the discontinuation of medication, and appeared to support participants’ increased self-agency and motivation in their personal recovery processes.

**Conclusion:**

Health care in Norway is perhaps one step closer to optimizing care for people with psychosis, allowing for more patient choice and improving the dialogue and hence the interpersonal relationship between the patient and the therapist. Personal patterns of suffering can be explored within a system aiming to support and have a higher level of acceptance for the discontinuation of medication. Such a system requires personal agency in the treatment regimen, with more focus on personal coping strategies and more personal responsibility for the recovery process.

## Background

In 2015, the Norwegian Health Minister, following the advice of user organizations, urged the four regional health authorities to offer medication-free treatment to persons experiencing psychosis [1]. In 2016, the Western Norway Regional Health Authority started integrating more psychosocial interventions into existing mental health care services in district psychiatric centers to comply with this guideline. The provision of increased psychosocial intervention options within mental health care was intended to enable patients wishing to discontinue medication to do so in a supportive setting. This change in mental health care emerged from the debate on the use of antipsychotic medication (referred to as AP medication) as a part of the treatment for severe psychiatric illness [2]. On the one hand, AP medication is recommended in the short term to reduce positive psychotic symptoms and in the long term to reduce the risk of relapse [3–5]. In some studies, AP medication has been associated with increased survival [6–8], and the discontinuation of AP medication has been associated with poor long-term outcome [9], including increased risk of violence [10, 11]. Severe mental illnesses, such as schizophrenia, have a substantial negative effect on life expectancy, together with an increased risk of suicide [12–14], which is also related to a lack of adherence to antipsychotic medication [15, 16]. The discontinuation of AP medication is often described as non-adherence rather than as an integrated part of a treatment regimen in collaboration with psychiatrists.

On the other hand, studies show that the dose reduction/discontinuation of AP medication is superior to maintenance treatment for long-term recovery [17, 18] and that the guided discontinuation of medication might be successful [17, 19]. Adverse effects of AP medication have been suggested to increase the risk of early death [20–24]. The debate raises important questions regarding treatment recommendations, and patients need to consider potential benefits as well as adverse effects when deciding whether to use AP medication [25–28].

The introduction of optional medication-free treatment for psychosis is a recovery-based reform of mental care based on advocacy work by service user organizations. The global recovery movement works to change mental health policy and practice based on the perspectives of people with mental illnesses. It has roots in both user organizations and wider civil society [29]. Qualitative studies and meta-syntheses have shown the importance of aspects beyond symptom reduction for the recovery process. Such aspects include interpersonal processes, increased understanding of one’s own pattern of suffering, and increased hope and motivation, which lead to self-agency in the treatment process [29–32]. There are relatively few studies focusing on the first-person perspective in the implementation of new treatment programmes in mental health care [33], and to our knowledge, no studies with personal accounts of treatment programmes integrated in existing services aiming to support patients in choosing to discontinue antipsychotic medication have been published.

We believe there is a need to explore whether aspects known to be important for the recovery process are present in users’ descriptions of their treatment experiences within the medication-free programme in Bergen, Western Norway. Hence, this study aims to use qualitative methods to investigate the experience of recovery following new treatment options and choices.

## Methods

### Site

The Norwegian health system is largely a public health system. It is organized into four regional health authorities, which each chose different approaches for the implementation of the medication-free treatment programme. The Western Norway Regional Health Authority aimed to improve health care for all patients with psychosis by tailoring treatment to individual preferences and integrating more evidence-based psychosocial interventions into existing services in district psychiatric centers. The treatment options offered were individual psychotherapy including cognitive therapy, Illness Management and Recovery (IMR) groups, individual job support (IPS), music therapy, and physiotherapy, including various groups for exercise. The services were designed to support whichever choice the patient made regarding both medication and psychosocial options. The focus was on increasing users’ involvement and sense of ownership of therapy, as well as improving the patient-therapist alliance. The services were not designed to promote one treatment choice over another. The medication-free project established a website with information [34] and held a conference as well as local seminars at the different clinics to inform staff. All patients who are above 18 years old, not restricted by coercive measurements, and within the admission area are eligible for medication-free services.

### Design

This was a qualitative study that included semi-structured, in-depth interviews (topic guide available, see Additional file 1) with people with psychosis who were registered for medication-free treatment. Qualitative methods such as in-depth interviews aim at understanding and representing the experiences of people as they encounter, engage, and live through situations [35–37]. This study also employed a service user-involved approach [30, 38] developed within a hermeneutic-phenomenological epistemology. Following this approach, the research team had a phenomenological aim to explore and describe the lived experiences of personal recovery processes within mental health care where medication-free treatment for psychosis has been proposed. Further, the co-authors recognize that our attempts to adopt such an approach inevitably involved interpretations.

### Researchers and user involvement

The first author has no health professional background, which was preferred by our collaborating user organization. Together with the first author, the supervisors and co-authors of this article constituted an interdisciplinary research team including a professor in music therapy, an associate professor in psychology, a professor in psychiatry, and a professor in medicine.

To ensure respect for the complexity of users’ views on the topic of this study, the research team invited four experts by experience to be co-researchers on this project. Three of these co-researchers are members of the user organization “Hvite Ørn,” and the fourth works as peer support staff. They were involved in the study from the preparatory phases (developing the protocol and research questions and discussing the semi-structured interview guide) through the data analytic phases (participating in the team-based analysis) and the dissemination of the results (participating in writing articles and presenting the research project). Studies have shown user involvement to be useful in improving research questions, ensuring that interventions remain “user friendly,” and improving the selection of outcome measures [39]. The authors believe this involvement enhanced the quality of the study through the development of a meta-perspective on the research process [30, 38, 40].

### Procedures

The protocol for this study was developed in collaboration with the user organization and supervisors. The semi-structured interview guide (supplementary file) was also a result of a close collaboration between co-researchers and the supervisors, as well as the first author. The interview guide covered four main topics: the participants’ life stories, their encounters with the health care system, their experiences of the freedom to choose, and their thoughts about the future. Within each of these topics, there were several open-ended questions and potential probes to elicit participants’ narratives of their experiences.

The first author conducted the interviews and made notes of her experiences after each interview to promote reflexivity and to be able to better remember the setting and ambiance of the interview at a later date. She obtained informed written consent from each participant to participate in the study and ensured the well-being of each participant after the interview. None of the participants expressed a need for further support. Eleven participants were interviewed during fall 2017 and spring 2018, and one was excluded from the analysis process for this article, as the informant had no intention of discontinuing medication. The interviews varied in length from approximately 45 to 90 min. All interviews were tape recorded and transcribed by the first author.

### Participants

The participants were people with psychosis registered as patients in one of two district psychiatric centers for mental health services in Health Bergen. Six participants were in a medication-free treatment course, while four had chosen to start medication again after having reduced or discontinued their medication in collaboration with their psychiatrist.

All participants were informed about the study by their therapists, orally and in writing. The therapists assessed eligibility for this study following the inclusion criteria of being above 18 years of age, being able to give informed consent, presenting with psychosis, and being a patient at one of the three district psychiatric centers. The participants also had to be actively engaged in medication-free services, which could be exercise, music therapy, job support, or other group therapy sessions.

The participants were purposefully selected to vary in age, gender, and past treatment histories to ensure diverse patient experiences (see Table 1 for details). There were five females and five males; nine were aged 25–40 and one 45–50. The number of admissions varied from 0 to 5 (6 participants) and 10–20 (3 participants), and one had 20–30 admissions. Age of introduction to psychiatric healthcare varied from 17 to 41. The participants could choose where they preferred to do the interview. Most chose to be interviewed at the district psychiatric center, in either the first author’s office, a quiet room or the room used for music therapy. One patient chose to be interviewed at home. In Table 1, the term “aborted medication free” means the patient had an intention to discontinue AP medication but decided to go back on AP medication for some reason and did not report any immediate intention to discontinue the medication again at the time of the interview. “Discontinued” means the patient had succeeded in discontinuing AP medication and did not express the intention of or need for using AP medication again at the time of the interview.

**Table 1:** Participant details

Patient	Diagnosis	Known medication, including previous and discontinued	Treatment at the time of the interview
P1	F20 Paranoid schizophrenia	Olanzapine long-acting injection	Aborted medication free, IMR, FACT, AFR, MI, psychotherapy.
P2	F20.3 Schizophrenia	Aripiprazole long-acting injection, Buprenorphine	Aborted medication free, AP medication, IPS, IMR, psychotherapy.
P3	F23.3 Acute paranoid psychosis	Olanzapine	Discontinued, medication free, psychotherapy.
P4	F29 Unspecified nonorganic psychosis	Aripiprazole, Quetiapine	Discontinued AP, music therapy, IMR, group therapy, psychotherapy
P5	F25.1 Schizoaffectiv disorder, depressive type	Aripiprazole, Lithium	Low dosage AP, music therapy, art therapy, ACT, psychotherapy.
P6	F23.9 Acute and transient psychosis	Escitalopram	Discontinued, medication free, psychotherapy.
P7	F25.1 Schizoaffectiv disorder, depressive type	Quetiapine	Discontinued, medication free, IPS, IMR, psychotherapy.
P8	F20.0 Paranoid schizophrenia	Aripiprazole, Sertraline	Aborted medication free, IMR, IPS, group therapy, psychotherapy.
P9	F41.9 Unspecified anxiety. Previously F22.0 Paranoid psychosis	Amisulpride	Discontinued, medication free, IMR, IPS, psychotherapy, exercise.
P10	F25 Schizoaffectiv disorder, manic type	Aripiprazole long-acting injection	Aborted medication free, excercise, FACT, psychotherapy.

Abbreviations: *IMR* Illness Management and Recovery; *IPS* Individual Placement and Support; *AP* Medication: Antipsychotic Medication; *ACT* Assertive Community Treatment; *FACT* Flexible Assertive Community Treatment; *MI* Motivational Interview

### Data analysis

The transcribed text was analysed using Attride-Stirling’s [41] thematic network approach. The text analysis was conducted as a team, with all co-authors being invited to read and comment on the raw, anonymized transcripts as well as be part of the coding process. Attride-Stirling’s thematic network analysis [41] provides procedures for conducting analysis of interview data, enabling the methodological systematization of textual data, facilitating the disclosure of each step in the analytic process, aiding the organization and presentation of the analysis, and allowing a sensitive and rich exploration of the structures and patterns of a text [41]. The first author (CO) performed the first coding together with two fellow PhD students who were not otherwise involved in the study, forming a coding framework and discussing the possible thematic network based on the first three interviews. This procedure is considered to strengthen the credibility of the chosen codes, as it enhances the rigour of the data analysis process. The codes emerged from the text, and CO, together with the fellow PhD students, identified the basic themes common across the interviews. After this initial coding, the coding framework was further developed as an iterative process with most co-authors collaborating and providing feedback. The basic themes were grouped based on their related conceptual content into the following organizing themes: “interpersonal relationships,” “patterns of suffering” and “motivation and personal agency in the recovery process.” The research team openly discussed inter-rater agreement and disagreement, taking care to emphasize the importance of the feedback from the experts by experience. This process also gave the co-authors the opportunity to provide information and understanding based on their various professional backgrounds. The themes were named and renamed for a better fit until the team felt the final product was representative of all views, and no essential information was lost in the process. The final global theme reflected the research question via the codes, basic themes and organizing themes. The translated coding frame relevant for this article is displayed in Table 2. The codes and themes, along with key quotes used to illustrate the findings, were translated into English by the first author. The research team used the NVivo software program for data management (NVivo qualitative data analysis software; QSR International Pty Ltd. Version 12 Plus).

**Table 2:** Relevant codes and themes from the analysis using Attride-Stirling’s thematic network analysis [41]

Codes	Basic themes	Organizing themes	Global theme
Information – treatment options and rights	Communication skills	Interpersonal relationships between therapists and patients	Personal recovery processes facilitated by more psychosocial treatment options within mental health care – medication-free treatment programme
Doctor, trust and availability			
Power play	Potential difficulties		
Substituting AP medication with other treatment			
The importance of having a choice	Processes of treatment choices	Patterns of suffering and how choices are made	
Choosing the unknown			
Choosing medication; effects, side effects			
Getting experience	Developing personal illness understanding, considering consequences		
Worsening: not an easy way out			
Outside factors, keep work and family			
Expectations; do it myself	Personal responsibility for recovery	Motivation and personal agency in the recovery process	
Coping strategies			
Doing stupid things			
Being independent, not telling	Future life hopes and thoughts, independence in life and treatment situations		
Dreams and hopes; work, studies, family			
Not being hard on myself			

### Ethics

The Regional Ethics Committee for Medical Health Research (REK southeast 2017/736) defined this study as health service research; hence, according to the Norwegian health research legislation, the study was approved by the local data protection officer. The data protection officer for Health Bergen approved the study in July 2017 (2017/8692).

## Results

The data analysis framework, from the codes to the global theme, is illustrated in Table 2. This results section is structured according to the organizing themes: interpersonal relationships between patients and therapists, the patient’s understanding of personal pattern of suffering, and personal motivation for self-agency in the recovery process.

### Interpersonal relationships between therapists and patients

When the participants in this project were asked about their reasoning for their choices, they expressed uncertainty both regarding the treatment options available to them and explanations for their choices. Answers such as “I’m not sure” and “I don’t remember” were quite common. One participant mentioned a lack of information regarding a patient’s rights to complain about the treatment:

P9: “She could have informed me better about my rights; if I disagreed with her. ( …) I had to figure that out by myself.”

Inadequate information included a lack of information, withheld information and an underestimated need for repeated information. Importantly, some participants reported that the type of service offered seemed rather arbitrary rather than a “real choice.” Not all services were available, and furthermore, not all services were suggested by the therapists:

P4: “I feel it’s kind of random which services you are offered and what you end up getting, really, and if you get a service that helps, in a way. But it is of course difficult to know what helps.”

Treatment choices were thus suggested to be limited in terms of availability and the information provided by the therapist, as well as the individual need for repeated information when illness and symptoms might affect memory [42]. This finding indicated a need for increased focus on shared decision making.

However, the level of information is not the only parameter of the quality of an interpersonal relationship that is considered important for the outcome of the therapy [43]. Trust is vital for therapy outcomes. In this study, the participants shared an overall feeling of confidence in their therapists. In response to questions about who they would trust to provide advice about their treatment choices, all participants mentioned their current therapist, along with other key persons in their lives. The availability of the therapist was closely linked with descriptions of a positive patient-therapist relationship. One participant described his psychiatrist as easy to reach, and he felt he could take part in decisions concerning his own treatment:

P7: “I really like that here. I can talk with (name) in the hallways, and if I have to schedule another appointment, or ( …). Sometimes we talk for ten minutes without having an appointment, and I get a new prescription and just talk. We do talk about different mood stabilizers and what he recommends and such. So, it might be that I will start a new medication again that I told him that I wanted to consider.”

Nevertheless, there were also some examples of distrust and not mentioning sensitive issues to avoid uncomfortable situations. Such uncomfortable situations could include talking about the worsening of symptoms or wishing to change or discontinue a medication. One participant described powerlessness in the relationship and talked about communication as a “game”:

P2: “So, I kind of picture that ‘NO’ ahead of me. And then I think, ‘Is it any use to bring it up? They decide.’ So, it’s kind of a game, I feel, where he has the power, and I don’t have much to say.”

Building trust could take time. Several participants described having had trust issues with the therapist or health care system in general, often linked to a period of worsening and their admission, but then being able to repair the relationship over time. One participant described this process:

P7: “Yes, well, he has been there quite long, through the worst of times, I mean … the psychiatrist. It’s quite special. Now, I think he is nice, but in the beginning, I didn’t think he was nice at all ( …) I didn’t like him.”

The participants reported that their illness and change in symptom severity could affect the experience of the quality of the relationship.

Despite the experienced trust, in regard to the process of the discontinuation of medication, the participants reported being presented with certain conditions. Therapists could accept their wish to discontinue AP medication, but not without substituting the medication with other treatment:

P5: “The impression I get is that I will be allowed to be psychotic if I want to, but then I have to do other stuff in order to maintain wellness in the psychoses. So, then she talked about music therapy and that it would be a good way to stay in therapy.”

In this way, therapists substituted medication with other available treatment options.

### Personal patterns of suffering and how choices are made

Participants in this study could choose between an increased number of treatment components, such as cognitive therapy, illness management and recovery (IMR) skills training, individual job placement and support (IPS), music therapy, exercise and family group therapy. All of the participants confirmed the importance of having a choice in their treatment when asked directly. One participant said,

P2: “It means so much for me to have a choice. Yes. To choose. To choose in psychiatry is incredibly important. And that they see possibilities. That it is not always that particular intervention, that one and only particular medication, you know! Because … they have to see the person in a wider perspective.”

Many of the mentioned services were unfamiliar to the participants, which made it hard to choose, both for the participants as well as for their family or peers:

P4: “I don’t know what they would have chosen for me. It’s hard to say. If you don’t completely understand, or if you don’t know exactly yourself, what actually helps.”

In this study, increased psychosocial intervention options within mental health care were intended to enable the discontinuation of medication in a supportive setting. However, quitting medication was not an easy way out in a life with illness. The participants in this study were all struggling with different medication issues. Many described the use of medication as characterized by fear of the unknown and adverse effects, as shown in the quote below:

P1: “But there is no definite answer to what happens when you are taking a pill. ( …) Because … then you might think all your problems are due to the medication. And then you think they will go away when the medicine is gone, and then you quit on your medication, and then they don’t go away.”

Thus, the participants recognized that taking medication is complex. Using medication may result in adverse effects, but discontinuing may not be an easy solution. One informant explained that he knew his delusions included delusions about medication, making him believe that the pills were poison and that the pain and aching in his body were severe adverse effects killing him. These delusions led to a wish to discontinue medication. In particular, forced medication was associated with delusions:

P7: “And … I don’t think I would have taken any medication if I just got forced to do it. I think I would have become very sceptical if I was … That is, I would have had delusions about it, being forced to take medications I did not think were good for me.”

Wishing to discontinue medication might have stemmed from delusions for some of the participants; however, the side effects from the use of AP medication must be recognized. Regarding the experienced side effects, some participants reported losing control over their body parts, one participant mentioned a feeling of drowning, and most participants talked about gaining weight and feeling tired:

P10: “I think it really sucks that I become more tired when I use that medication, and I also feel a bit like a failure when I use it. It’s like I have a defect.”

Patterns of suffering are individual, and gaining experience with the various effects that medication has on one’s body is a learning process. Not all participants wanted to reduce all symptoms of their illness; for example, one participant said,

P5: “Perphenazine works too well. It removes too much of the psychosis. When I’m psychotic, I’m more friendly. I get more … naïve? I become … they called it pronoid. I sort of haven’t completely said goodbye to the psychosis yet.”

Other participants also described a similar relationship with their symptoms, such as that hearing voices made them feel accompanied and that they felt lonely without them.

Four of the participants in this study had aborted the discontinuation of medication at the time of the interview. One informant described this experience and the process of learning what worked for him:

P8: “I think that someday, I can stop. ( …) But I know it is smart to use medication too. It sort of soothes the psychosis, so it makes it easier to cope and do stuff. So, the medication helps, no arguing there.”

Outside factors, such as having to work, were also important to consider in the participants’ processes of learning about their own patterns of suffering:

P10: “But I can’t risk getting ill again since I have a job now … So, I can’t risk losing my job … As long as I get just a little bit of Abilify, I’m safe. It might be that I could have coped on an even lower dose … we’ll see. I might consider that.”

The complex learning process involves becoming experienced with one’s own illness; the symptom load, the adverse effects, and the outside factors all contribute to decision making about treatment options.

### Motivation and personal agency in the recovery process

Recovery-oriented pathways require personal agency and involve a responsibility to improve one’s life. Several participants expressed a feeling of having to “do the work” themselves:

P2: “I have to do the work. I think a lot of people have helped me along the way; now, it’s just me who has to do the work. That’s how I feel. And I intend to do it.”

By having to “doing the work,” the participants meant they had to employ coping strategies such as avoiding excess stress; staying away from drugs; or maintaining a daily routine of sleeping, resting, and eating well. Taking responsibility for their well-being implied a risk of failure. Their coping strategies were challenged by their symptoms and illness. One informant described how the worsening of symptoms pushed away the care team so that they were unable to intervene:

P10: “What happened to me first was that I started to be a bit bitter toward psychiatry in general; I didn’t want anything to do with them (the care team) at all. So, I think it was a bit unfortunate they didn’t catch me at once, because I sent some messages to one of them … They didn’t know what to do, they said then. But I think it was quite unfortunate they didn’t catch on earlier that I was ill.”

Much of therapy involves learning how to live with the symptoms. Sometimes participants wanted to choose without help from others, relying on their own experience and expertise, as participant said:

P2: “So, I have been very determined to deal with all of this by myself. ( …) So, I have been very independent.”

When the need for independence involves not telling carers about one’s symptoms, there is a risk of the worsening of symptoms becoming out of control. Nevertheless, another outcome would be to increase the level of independent living. Both outcomes might offer valuable lessons in the process of recovery.

Many of the participants’ hopes for the future evolved around managing one day at the time. Some mentioned work, studies or perhaps having a family. One informant described her thoughts about her life:

P5: “Now, I just want to figure out everyday life and how to be around myself and be … in my own company … And have a good time with myself, be happy with who I am, and sort of … get a self-image that fits with reality and … not be so hard on myself as I have been.”

The same informant continued when asked where she saw herself in 10 years:

“I hope I’m not dead … No, I hope I’m alive, that’s the only thing I hope for. I can’t say I have any … I hope I’m ok. I would have loved to have a husband and family, but that’s kind of distant to me.”

This quote expresses the participant’s need to not be so “hard on herself” as a coping strategy, which is consistent with her understanding of her own vulnerability, as well as her fear of not surviving the illness. The task of both surviving psychosis and maintaining hope for a better future is demanding.

## Discussion

The integration of medication-free services into existing services has resulted in more treatment options for all persons with psychosis who are eligible for outpatient treatment in Bergen. The participants in this study shared a generally positive impression of their interpersonal relationships and communication with their current therapists. Developing trust with the therapist was said to depend on the level of symptoms as well as continuity in the relationship over time, and the relationships were described to have improved compared to previous experiences. These findings might indicate an increased effort that therapists have made to meet patients’ needs and present higher acceptance of patient choices. This result is in contrast to those of other studies on collaboration between therapists and patients [44–46]. However, potential difficulties that participants cited were a perceived lack of information about rights and treatment options available, as well as some avoidance of sensitive topics in the therapeutic dialogue. According to a Norwegian report on outpatient clinics in 2007, users reported a need to improve the level of information on available treatment options [47]. It seems there is still room for improvement in information flow. A digital tool for shared decision making for people with psychosis was developed in 2018–2019 and was launched in August 2019 to be implemented in the Western Norway Health Region to improve these issues [48].

The process of choosing treatment was described as complex with many influencing factors. Each person shared individual stories displaying a reflexive understanding of their individual strengths and vulnerabilities linked with increasing understanding of the illness, including considerations of potential consequences of worsening symptoms. Studies of health care decision making have shown that patient choices seldom are based on reasoning alone. Elements such as trust, intuition, emotion and beliefs also matter [49]. This is in line with the present study findings, which showed that factors influencing treatment choices, particularly those regarding medication, included a fear of the unknown, delusions, “not knowing what helps,” and the beneficial aspects of symptoms. One of the participants shared that she felt defeated by having to take pills for an illness in the brain, as if she had a physical defect. Some studies have suggested that having to use medication for a mental illness may be stigmatizing [50], but the participants did not otherwise mention stigma surrounding psychiatric illness as much as expected, even when the first author probed on this topic during the interviews.

Personal responsibility and motivation for the recovery process was highlighted by most of the participants, often associated with an extensive focus on coping strategies. The participants generally concluded that they “need to do the work on their own” in their recovery processes. They regarded their coping strategies as important tools to keep their symptoms under control. Several of the implemented treatment options focus largely on coping strategies. The emphasis on coping strategies needs to be closely monitored, as studies have shown significant associations between self-stigma and coping strategies in schizophrenia [51, 52].

Thoughts about the future included both hope for independent living as well as a certain resignation to facing life with an illness. The learning processes that resulted from the choices participants had made sometimes came with a cost. Four participants had aborted the discontinuation of medication, as they were not able to cope with the symptoms without medication, with some experiencing adverse events as a result. Others felt they coped well and were satisfied with a life with lower dosages of AP medication or without AP medication. These findings show how increased psychosocial intervention options support personal recovery processes such as increased self-agency and motivation, which is in line with findings from other studies [53, 54]. However, it is important to take into consideration the possibility of risk related to both the discontinuation process and potential self-stigma in the use of coping strategies.

This study has strengths and limitations. It had a limited number of participants, and the interviews were performed soon after the implementation of the treatment programme had commenced. Therefore, the health system and services may not have been fully acquainted with the change at the time of the interviews. However, this study provides first-person perspectives on choosing treatment within a health care system undergoing change through the implementation of more recovery-oriented treatment options. It is important that context-specific users’ perspectives are considered in the research on the implementation of new treatment programmes.

## Conclusions

Health care in Norway is perhaps one step closer to optimizing care for people with psychosis, allowing for more patient choice and improving the dialogue and hence the interpersonal relationship between the patient and the carer. Within a more supportive system, personal patterns of suffering can be explored in relation to factors that are known to facilitate personal recovery. Such a system demands a higher level of personal agency in the treatment regimen, more focus on personal coping strategies and more personal responsibility for the recovery process.

Clinical implications from this study include the recommendation of an increased level of psychosocial interventions and shared decision making in mental health care that are adapted based on the level of symptoms, experience and individual preferences. Additionally, it is important to take into consideration the importance of continuity over time in developing interpersonal relationships between patients and therapists.

## Supplementary information

**Additional file 1.** Short topic guide used for the in-depth interviews with the patients, translated from Norwegian.

## Data Availability

The dataset that supports the findings of this study consists of in-depth qualitative patient interviews, which are not publicly available for confidentiality reasons. The entire coding framework developed from these interviews is available from the corresponding author on reasonable request. The interview guide is available as a supplementary file.

## Abbreviations

AP Medication: Antipsychotic medication
IMR: Illness management and recovery
IPS: Individual placement and support
ACT: Assertive community treatment
FACT: Flexible assertive community treatment
MI: Motivational interview
