# Supplementary material for: “It means so much for me to have a choice”: a qualitative study providing first-person perspectives on medication-free treatment in mental health care
Source: BMC Psychiatry. 2020 Aug 8;20:399. doi: 10.1186/s12888-020-02770-2 (PMC7414551; doi:10.1186/s12888-020-02770-2)
Supplement: Supplementary file 1 — Additional file 1. Short topic guide used for the in-depth interviews with the patients, translated from Norwegian. [file 12888_2020_2770_MOESM1_ESM.docx]

Interview guide – for patients

Focus is the patient’s personal story about their life and illness, and the choices they have made along the way. Process orientated, focus on the course of the illness and general background.

Follow-up questions:

Can you tell me about your experience with having this illness?

How do you experience being psychotic?

How did you first know you were ill? Can you tell me about the first time you were psychotic?

What is most important to you when you feel ill?

To you, what does non-medical treatment mean?

Why did you choose not to use medication in your treatment?

How did you learn about the non-medication treatment option?

How do you think your therapists consider this option?

Are there any differences between the therapists when it comes to attitudes towards medication?

Do you feel your choice about non-medical treatment is a free and independent choice?

What, if anything, influenced your choice of treatment?
